# Coaches’ Perceptions of Common Planning Concepts Within Training Theory: An International Survey

**DOI:** 10.1186/s40798-023-00657-6

**Published:** 2023-11-21

**Authors:** Kechi Anyadike-Danes, Lars Donath, John Kiely

**Affiliations:** 1 Department of Intervention Research in Exercise Training, German Sport University Cologne, Cologne, Germany; 2 Faculty of Education and Health Sciences, University of Limerick, Limerick, Ireland

**Keywords:** Periodization, Resistance training, Inter-individual response, Coaches’ perceptions

## Abstract

**Background:**

The planning of training is a popular yet controversial topic among coaches and sports scientists. Periodisation is often presented in the literature as the most efficacious approach to planning training. While historically surveys of coaches appeared to support this a key failing was that no unified definition of periodisation exists. Recent surveys offering a periodisation definition and an alternative planning methodology found many choosing the alternative therefore questioning periodisation’s wide acceptance. The current survey looked to explore how coaches perceived specific concepts, drawn from the literature, that relate to the planning of training.

**Methods:**

106 coaches [age range: 18–65+ years, 31% 15+ years coaching, 58% individual-events/sports and 32% international level] from across the world completed a novel cross-sectional online survey on the planning of training and the training process. Topics included use of periodisation, division of time into discrete periods, assignment of goals and training to pre-determined periods and the adaptability of pre-established plans.

**Results:**

The majority described their planning approach as training periodisation (71%). Similarly, there was strong agreement with the necessity to determining a goal for the season (85%) and divide the season into distinct manageable periods of time (73%). When examining whether physical adaptations are achievable within specific and fixed timeframes only a minority (33%) agreed, a similar result was found for training physical capacities in a sequential order (37%). Finally, there was limited support for training targets remaining fixed over a training period (10%).

**Conclusions:**

As a tool for the planning of athlete’s training, periodisation is often presented as the best and most popular approach. Recent research however has highlighted possible discrepancies in its usage among practitioners. The results of this survey echo this and question the acceptance of periodisation concepts even among periodisation users. In part this may be due to key tenets of periodisation no longer being supported by research or practice. A lingering question then is whether the beliefs of coaches, developed through experience and supported by research, will continue to be marginalized. If sports scientists wish to aid coaches then they need to be engaged in future research initiatives as co-collaborators.

**Supplementary Information:**

The online version contains supplementary material available at 10.1186/s40798-023-00657-6.

## Key points

- Many coaches, even self-described periodisation users, did not employ key periodisation concepts such as training specific fitness factors in a sequential order.
- Rather than periodisation, many coaches’ practices seemed to align with what could be considered planning best practice, based on the levels of uncertainty in the training process.
- Given the lack of periodisation alternatives in the literature, and the results of this study, future research should explore how non-periodisation coaches formulate, manage and deliver training plans.

**Supplementary Information:**

The online version contains supplementary material available at 10.1186/s40798-023-00657-6.

## Background

The idea of planning training for increased performance is a popular though controversial topic that has a long history with many different approaches having been documented [1]. The approach frequently featured in the scientific literature is periodisation. Indeed a recent literature review stated that a “periodized training process is considered the *principal* planning strategy for athlete development and preparation by *most coaches and sport scientists*.” [1].

The term periodisation as it relates to athlete preparation and the planning of training can be traced back to 1960’s USSR where it was coined by sports scientist Lev Matveev [2]. While the planning of athletes training was nothing new Matveev was posed the question of “How to peak at the right time?”. A perceived problem for the USSR was the inability to guarantee that their athletes would experience their best performance when needed. Based upon his research findings and the incorporation of then contemporary research he proposed periodisation, an approach based on science that would ensure an athlete experienced their peak performance when planned [2].

It is important to note however that objections existed from the start to periodisation’s superiority and wide applicability [2]. Due to its original conception being for sports with short seasons and few peaks it was not deemed universally applicable, with team sports noting specific problems [2]. Within scientific literature things are complicated by the lack of a unified definition for periodisation with one review finding at least 80 in the literature [3]. Recently it was described as “the *macromanagement of the training process* with respect to time. In other words, *time is allocated toward various fitness phases that are strategically aligned* in a unilateral fashion toward competition.”[4]. Through this lens periodisation is an exercise in planning and management, in-particular macro-management. In contrast programming (the micro-management) deals with training specifics (e.g., exercise selection) [4]. Using definitions in the peer-reviewed literature Kataoka et al. proposed that “periodization *divides the training plan into discrete* cycles, phases, or blocks that focus on developing *specific physiological adaptations* (i.e., muscle hypertrophy, strength, power, speed, aerobic endurance, and others). Over time, the training program will typically *progress from general to specific* adaptations with the intention of bringing *peak performance at competition time*” [3]. These definitions highlight the training process being broken into smaller periods which are sequenced in a predetermined order, each period lays the foundation for the next in a “mutually dependent” nature [1, 5]. Furthermore, in periodisation “*once planned, fitness phases* and other respective timelines are *largely static*”, though specific training parameters may vary [4].

Previously a review of surveys examining different practices among strength and conditioning coaches (SCC) seemed to support periodisation’s dominance with 89% indicating they used it [6]. Indeed, one such survey of SCC working in professional soccer reported that 98% used periodisation [7]. However, a recent survey of SCC working in elite Brazilian soccer found that only 6% indicated that they used periodization [8]. This stands in contrast to both the results of the review and the prior survey of SCC in professional soccer [7]. A possible reason for this divergence is that earlier surveys simply asked coaches whether they used periodisation, a problem considering the lack of consensus for a definition. In contrast the survey of Brazilian coaches gave descriptors for two different planning approaches [8]. The first gave a description of periodisation highlighting pre-planned or fixed routines with players peaking for matches deemed most important (6%). The second descriptor detailed programs that were frequently readjusted according to physical and physiological responses with the purpose being to maintain high levels of performance (94%) [8]. While similar findings were also found among Argentinian rugby SCC an obvious question might be whether such an outlook is restricted to team sports given that periodisation was designed for individual sports such as track and field [9]. However, a survey of Brazilian sprint and jump coaches reported fewer chose periodisation over the ‘flexible’ alternative (31.6% vs 52.6%) [10]. These surveys highlight the issue of not providing a formal periodisation definition and suggest that key aspects of periodisation might not be seen as useful among all coaches.

As a tool periodisation is actively promoted not just in the peer-reviewed literature but also within the education space. An examination of the course description for a range of organizations offering SCC certifications shows that they all have time spent teaching periodisation [5, 11–14]. Furthermore, university degrees that offer cross accreditations for its students with these organizations need to cover periodisation. This is merely mentioned to highlight the pervasive nature with which periodisation seems to be taught, at least in an English-speaking context. However, while periodisation might sometimes be seen as synonymous with planning, it is in fact only but one approach as highlighted by the survey results and elsewhere in the literature [8–10, 15]. Due to a lack of research, it is also not clear whether national and/or sporting culture affects opinions on planning including periodisation usage. For example, the two surveys with low periodisation usage were performed in South America. Further as a possible reason for the lack of periodisation use they highlighted the “congested and demanding training and competitive schedules faced by team-sport athletes” along with high levels of performance needed most of the year [8, 9].

Therefore, though most surveys suggest that periodisation is the preeminent planning methodology there are potential caveats, specifically concerning the definition of periodisation given and the inclusion of a viable alternative. An alternative approach then is to ask coaches specifically about the core concepts that are reported within the literature to underpin periodisation [9]. This would allow for a more detailed understanding of coaches’ opinions and practices as well as whether theory and practice are aligned.

Hence, the objective of this survey was to describe how coaches (sports-specific and SCC) from a range of backgrounds view concepts, drawn from the literature, that relate to the planning of training. Due to its influence many of these concepts relate to periodisation and are considered fundamental to it. The surveys responses are then compared to the literature allowing for (1) an understanding of whether the meaning of periodisation is as varied among coaches as it is in the literature (2) among self-described periodisation users what concepts, if any, they use and (3) how do non-periodisation users respond to periodisation concepts.

## Methods

### Sample Selection and Administration

The survey utilized a purposive convenient sample due to the lack of a centralized coaching database needed for probability sampling. Participation was voluntary and all those who took part were notified they could withdraw at any point. Due to the fundamental nature of the topics explored within the survey there was a limited inclusion criteria of currently working with athletes as a coach (sports-specific or SCC), being at least 18 years old and English literate. With no agreed way to determine sample size for surveys we established ours based on similar studies leading to a minimum sample of 100 [6, 16–18]. The survey was available online through Microsoft Forms from November 2021 to February 2022. It was distributed through the authors social media accounts and personal networks. Though this approach has limitations (which will be discussed later) it is not uncommon [19–21]. At the surveys landing page potential participants could access an information sheet for the study. Participation required coaches to indicate that they had read the information sheet and gave consent.

### Study Design and Survey Development

It was determined to use cross-sectional study approach. A review of the English literature highlighted that no current survey featured detailed questions on the specific topics that were of interest. Therefore, it was decided that a new survey was needed. Recent surveys in the literature as well as texts on methodology were consulted to aid in the determination of best practice regarding reliability and validity [9, 18, 20–25]. Given the exploratory nature of the survey face/content validity were considered most important. This was in-turn combined with the piloting process which took place across two rounds with two separate groups. The first was with a small group of experienced coaching practitioners qualified to doctoral level (*n* = 3). The groups coaching experience was in a range of sports, including both team and individual events, and at a variety of levels simultaneously they were also still actively publishing within sports science. This round helped determine whether the survey accurately reflected the relevant literature, with feedback being used to improve content and clarity. A few examples of this included inclusion of questions, restructuring of questions and removal of questions (e.g., due to repetition, lack of precision). A second round of content analysis combined with piloting was then performed focusing on expression of concepts and clarity. For this a separate group of practitioners representative of the target population was used (*n* = 7). This feedback allowed for minor alterations and refinement. Other forms of validity such as concurrent, predictive and construct validity were not deemed appropriate. Regarding reliability as this survey was both exploring what coaches do and think, something that can naturally fluctuate over time, stability (test–retest reliability) was not considered necessary [16]. Internal reliability and inter-observer consistency were also not seen as appropriate.

The final survey was constructed around three distinct topics that ordinarily would be covered in distinctly separate surveys: (1) factors driving physical training adaptation, (2) ‘fundamentals’ of planning training and (3) the ability to predict training adaptations. This merging was done due to issues around recruitment and retention of participants. At the outset it was determined that these separate topics would be merged into one survey but then be separated back out for analysis. In part this was also considered necessary as it would not be possible to coherently cover all the topics in a single article. The authors felt that these circumstances met the criteria set out by the APA for separating a single data set into multiple publications [26]. This article deals with the second topic, that of the ‘fundamentals’ of planning training.

The questions discussed are available in Additional file 2 but are also given within each figure as they were presented to participants. Before being presented with questions regarding the key topics participants were asked to provide demographic information via nine questions. The questions in the survey were a mixture of single-item response variables, multiple response variables and ordinal scales. For the ordinal scales five-point options were used as it has been suggested that this number maximizes discrimination without sacrificing reliability with longer scales leading to data quality reduction [27]. Before submitting the survey participants were asked if they were willing to take part in planned follow-up questionnaires. Ethical approval for the survey was obtained from the German Sport University Cologne ethics committee. Additional information can be found in the Checklist for Reporting Results of Internet E-Surveys (CHERRIES) available in Additional file 1: Table S1 [28].

### Statistical Analyses

Due to convenient sampling only descriptive statistics (in the form of percentages) are presented as generalizations or inferences could not be made to the wider population [29]. Survey responses were exported to Microsoft Excel [30], anonymized, missing data checks performed and then explored in comparison with the literature. After an initial comparison to the literature the data set was split based on those who identified as using periodization and those not. While it would be desirable to make comparisons based on other factors (e.g., experience), or try to determine factors of those who do and do not use periodization, this was deemed inappropriate. This is due to the fact that neither correlations nor causation can be established using a non-probability sample.

## Results

### Background Information

On close 106 coaches had completed the survey with a further two accessing but not granting consent. The demographic details of the participants are seen in Table 1. Participants were predominantly male (92%) with a high level of formal education (60% postgraduate). The majority (84%) held a coaching qualification. Participants worked with team and/or individual sports at a variety of levels.

**Table 1:** Descriptive Characteristics of Coaches

Gender	Age	Academic qualification	Location	Coaching qualification?
Male (92%)	18–34 (38%)	School leaving qualifications (8%)	UK/Ireland (36%)	Yes (84%)
Female (8%)	35–54 (52%)	Bachelor’s degree (31%)	Europe (not incl. the UK or Ireland) (23%)	No (16%)
	55–64 (8%)	Masters degree (49%)	North America (23%)	
	65+ (3%)	Doctoral degree (11%)	Asia (4%)	
			South America (4%)	
			Africa (0%)	
			Oceania (11%)	

Years coaching	Individual or teams sports	Personal participation in sport	Level of athlete(s)
1–5 (20%)	Team (42%)	Yes (92%)	Amateur/Recreational (14%)
6–10 (29%)	Individual (58%)	No (8%)	Regional (25%)
11–15 (20%)			National (29%)
15+ (31%)			International (32%)

### Periodization Use

**Fig. 1:**
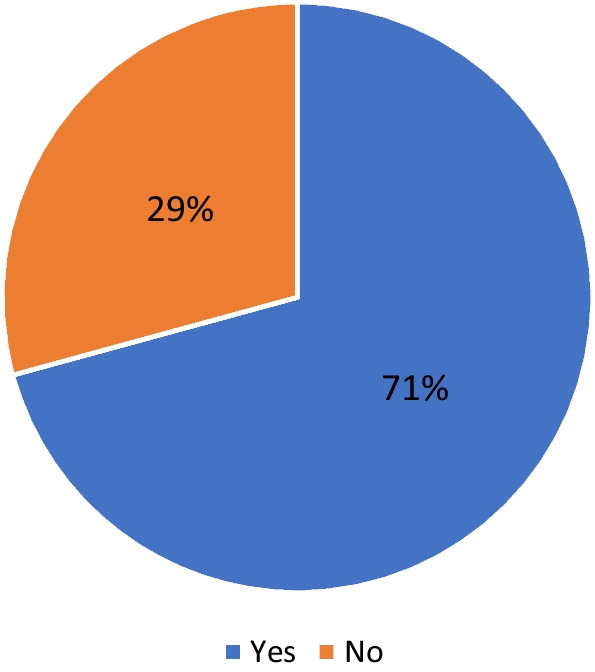
Percentage of coaches who described their approach as periodization (Would you describe your planning approach as training periodization?)

**Fig. 2:**
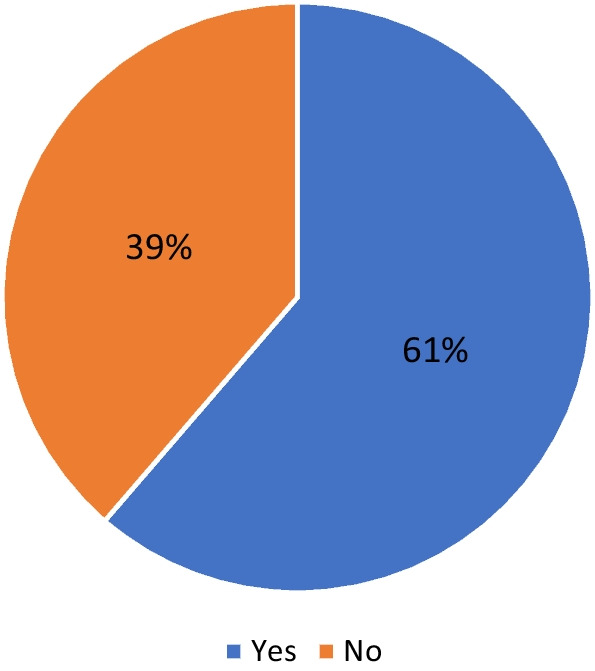
Perceived difference between periodization and planning (Do you see a distinction between periodization and planning?)

As indicated in Fig. 1 71% of the coaches described their planning approach as periodization while 61% saw a difference between periodization and planning (Fig. 2).

### Planning Fundamentals

**Fig. 3:**
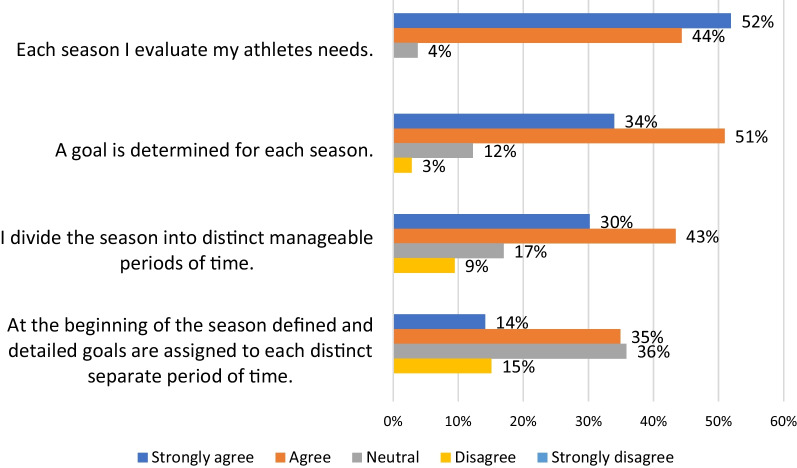
Responses to statements regarding fundamental planning concepts

Figure 3 shows responses to statements regarding fundamental planning concepts. When asked whether they evaluate athletes needs at the beginning of each season the overwhelming majority agreed to some extent (96%). A similar picture emerged regarding the setting of goals for each season (85%). The third statement was regarding the division of the season into distinct manageable periods of time which while the majority supported was less than the previous two (73%). Finally, almost half agreed (49%) that at the beginning of the season defined and detailed goals should be assigned to each training period. However, the single largest response was neutral (36%).

### Order of Training

**Fig. 4:**
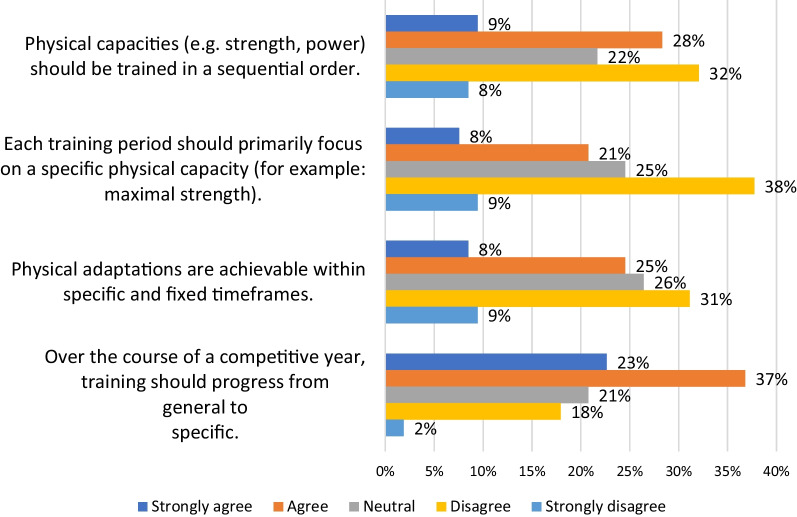
Responses to statements regarding the ordering of training periods

Figure 4 shows responses to statements regarding the ordering of training. Many of the coaches indicated to some degree (60%) that training should progress throughout the year from general to specific. Regarding whether physical achievements were achievable within a specific and fixed timeframe there was greater disagreement (40%) than agreement (33%). An increased number disagreed (47%) that each training period should primarily focus on a specific physical capacity though 29% did agree to some extent. A final statement about the need for physical capacities to be trained in a sequential order found more disagreeing (40%) than agreeing (37%).

### Revising the Plan

**Fig. 5:**
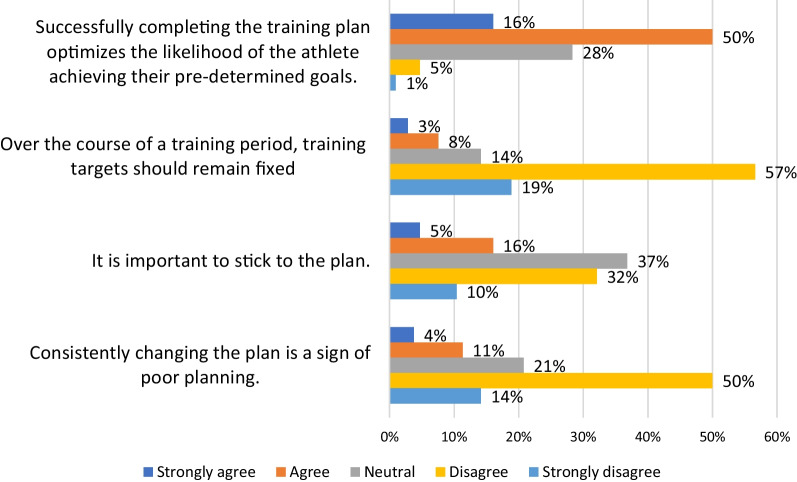
Responses to statements regarding the revising of the plan once started

In the final section coaches were shown statements regarding the revising of the training plan with responses presented in Fig. 5. Over half of coaches (66%) indicated that completing the training plan increased the likelihood that an athlete would achieve their pre-determined goals. When asked whether training targets should remain fixed over a training period, 76% indicated they disagreed with only 11% agreeing. Similarly, more coaches disagreed (64%) that consistently changing the plan was a sign of poor planning. Finally, when asked about the importance of sticking to the plan 42% disagreed while 21% agreed.

## Discussion

Within the literature there are ongoing debates over the utility of planning approaches like periodization in part due to issues surrounding efficacy [31, 32]. Furthermore, with a lack of agreement on a definition for periodization it has been difficult to determine its usage within coaching communities [3]. Therefore, our survey aimed to describe how coaches view a variety of planning concepts drawn from the literature many of which are fundamental to periodization. In alignment with prior surveys the majority of the coaches identified their planning approach as periodization (71%). However, just over half (52%) of these self-identified periodization users saw a distinction between planning and periodization. Among many of the coaches there was a general agreement about the use of some basic planning strategies which, though they occur in periodization, are not unique to. An example being the determination of goals for each season which the majority agreed to (85%). However, some concepts considered specific to periodization, such as training physical capacities in a specific order, had limited agreement (37%) with more disagreeing (40%). Perhaps most surprisingly, is that out of four statements reflecting specific periodization concepts only one, training proceeding from general to specific, was agreed upon by more than half of the periodization users. While superficially these results support the notion that periodization is the principal strategy used by coaches, as the adage goes the devil is in the detail. A more nuanced examination of the results highlights that though a coach may say they use periodization they in fact might not agree with key concepts outlined in the literature. A possible reason for many indicating that they use periodization is the lack of discrimination between it and planning within the bulk of the literature [15]. To best explore the results of this survey and for clarity’s sake the discussion is divided into three primary themes: (1) planning fundamentals, (2) order of training and (3) revising the plan.

### Periodization Use

Previous coaching surveys suggest that periodization is the principal term chosen to self-describe the methodology used for devising athletic training plans [18, 33–38]. Our survey reported similar findings, with 71% of coaches indicating they describe their training plans as periodized. However, given the variety of periodization definitions within the literature, it is difficult to know whether this is because planning and periodization are often conflated. When asked 61% of the coaches agreed there was a distinction between the two though over a third did not. However, it is worth noting that among periodization users just over half (52%) saw a distinction, a sharp contrast to non-periodization users (84%). There is a possibility that due to the presentation of periodization as the principal strategy, and the ambiguity around what it is, many coaches do not see a distinction. If true this is problematic as periodization is but one approach to planning. Indeed as highlighted by Afonso et al. “planning is the macro-level concept and *periodization is at an optional level* between general planning and programming.” [15].

### Planning Fundamentals

The first theme relates to coaches’ views on the initial planning process. An initial needs analysis, whereby key targets for intervention are identified, is conventionally recognized as a key component of the planning process [5, 39, 40]. In this survey the overwhelming majority of coaches agreed that athlete’s needs should be evaluated at the beginning of each season (96%). Similarly, the majority indicated that goals should be determined for each season (85%).

Fundamental to periodization is the division of a larger timeframe into shorter more discrete periods which are established by working backwards from the endpoint [3, 15]. In this study 74% of coaches agreed that they divided the season into distinct manageable periods of time. Given that only 71% of coaches indicated they used periodization it seems that for some coaches this tactic is not unique to periodization. Surprisingly, and further adding to the confusion about what periodization is, only 83% of self-identified periodization users agreed with this statement. Given periodization is frequently defined by the division of time into smaller periods this begs the question what periodization means to these coaches [41].

Finally, coaches were asked about assigning pre-determined goals to each distinct separate period of time something that is considered core to periodization [3]. The form that these goals take may differ due to the approach chosen with one example being the improvement of specific physiological adaptations [1, 4]. Among these coaches less than half (49%) agreed with the statement which is surprising given the number of periodization users. In fact despite the importance within periodization of pre-assigning goals to each period only 56% of periodization users agreed with the statement.

In summary, these results suggest many of the coaches are simply applying best practice with regards to planning. While dividing the season into distinct manageable periods of time is associated with periodization it is not the sole defining characteristic. It is the pre-assigning of precise goals to these periods, which many disagreed with, that might separate periodization from other planning approaches. This hints to the idea that since the needs of athletes and their competitive schedules can evolve over time then so should their goals.

Taking a wider perspective periodization resembles traditional project planning [42]. This approach is predicated on establishing and accounting for all influencing factors, with the belief they will remain constant throughout. In turn the exact time needed to accomplish each sequential objective can be calculated. While ideal this is not how many projects proceed in real life due to unforeseen and unknown factors [43]. Similarly due to the complex nature of humans this is also not how we adapt, rather it is better understood through nonlinear dynamics and the process of emergence [15, 31]. A key feature being that changes in input may produce disproportionate changes in output invariably leading to issues with predictability.

### Order of Training

This theme explored coaches’ views of core periodization theory tenets. As highlighted by Kataoka et al. and Stone et al. periodization definitions, each period of time (e.g., phase) should focus on developing specific physiological adaptations or fitness characteristics [1, 3]. For the coaches in this study the concept of focusing primarily on a physiological capacity (as advocated in block periodization for example [1]) was not overwhelmingly supported (47% disagreed). Even among the periodization users there was limited agreement for this concept (36%) with a large disagreement found among non-periodization users (74%). Notably, although this idea is fundamental to specific periodization approaches, there is little research supporting its efficacy either via training studies or underpinning theoretical work [1, 44].

An additional, and fundamental, periodization guideline is that physiological capacities should be developed in a specific and sequential order [1, 4]. Within the relevant literature, however, there is no definitive consensus on how these specifically focused phases should be ordered [1, 4]. When presented with a statement asking whether physical capacities should be trained in a sequential order, only 38% of the surveyed coaches agreed (45% of periodization users). Similarly, the majority of coaches surveyed by Loturco et al. and Zabaloy et al. indicated that the approach they used was one that was constantly readjusting to better align with either the individual or groups responses rather than one that was preplanned or fixed (94% and 69%) [8, 9].

A further implicit assumption, inherent in the periodization literature, is that the timeframes necessary to realize specific physical and/or performance adaptations are predictable [4, 15]. The need for this is highlighted in, for example, the Essentials of Strength Training and Conditioning, the primary resource for the preparation of the Certified Strength and Conditioning Specialist exam [45]. Within this text periodization planning is described as the process of partitioning training into “*mutually dependent periods of time* in order to induce specific physiological adaptations that underpin performance outcomes.”[5]. Despite this fundamental assumption of periodization, only 33% of coaches agreed that physical adaptations are achievable within specific and fixed time frames. Even among periodization users, more disagreed (39%) than agreed (36%).

This disparity between the coaches’ perspectives and the periodization literature exists also between academic periodization literature and contemporary training research. In contrast to periodization’s belief in predictable training outcomes, or time needed to realize them, contemporary evidence suggests that athletes adapt to similar training interventions at different rates and with different resultant magnitudes [46–48]. This extensive inter-individual response variability is exemplified by the results of Marsh et al. study where pairs of monozygotic twins responded differently when given the same type of training [49]. The authors highlighted that this is likely due environmental factors having a stronger influence than genetics. Given approaches such as periodization require all factors to be taken into account to make accurate predictions, the effect of environmental factors poses a serious challenge for this calculation.

In their synthesized definition Kataoka et al. mention that training should progress from general to specific adaptations [3]. Notably, however, the terms ‘general’ and ‘specific’, as represented in the literature, remain vague. For example one approach is linked to that of the work of Matveev's where the terms general and specific were in reference to motor abilities or sport skill while the definition given by Kataoka et al. is based on physiological adaptations [50]. When presented with the statement “Over the course of a competitive year, training should progress from general to specific.” coaches predominantly agreed (60%). There was, however, a notable differentiation between periodization (72%) and non-periodization users (29%). Given the possibility for multiple interpretations of the statement research should explore what this concept means to coaches and how it interacts with others such as not predetermining goals for each period.

The results of this section suggests that among these coaches the majority do not believe that physical training adaptations are achievable within specific and fixed timeframes. In the wider literature pre-planned approaches analogous to periodization are not recommended given the levels of uncertainty involved [42]. The training process has relatively high levels of uncertainty due to the limitations of predictability surrounding how an athlete will adapt to training or the concomitant timeline. This is evident when examining inter and intra individual changes in response to the same training protocol [51, 52]. In practice this means an athletes response to the same training ‘block’ has varying degrees of uncertainty, a serious problem for approaches like periodization where predictability is crucial [15]. This problem could in part explain the results found in this survey which align with others in the literature [8–10].

In summary, a core part of periodization is knowing the time required for specific adaptations an idea that can be traced to Matveev's research on peaking. Theoretically this allows for a coach to determine exactly how much time an athlete needs to adequately develop each attribute. However, if physical adaptations are not achievable within these timeframes then periodization faces fundamental problems. As implied in other studies it appears that many of the coaches surveyed question this fundamental tenet. As periodization was constructed to aid coaches if they no longer find it, as described in the literature, applicable then researchers need to understand why and seek alternatives.

### Revising the Plan

As previously noted by Cunanan et al. a hallmark of periodization is that “once planned, fitness phases and other respective timelines are largely static” [4]. Whether this perspective is universally shared among the coaching community, however, remains unclear. Accordingly, this section explores coaches’ perspectives relating to the adaptability of training plans once established.

When asked whether the completion of the plan optimized the likelihood of the athlete achieving their goals, 66% of coaches agreed, with only 6% disagreeing. This response, as it aligns with conventional planning perspectives, is perhaps unsurprising. Yet, interestingly, when asked whether it is important to stick to the plan only 21% agreed, even among periodization users more disagreed than agreed (37% vs 24%). These results echo those of three recent surveys where more coaches chose as their planning approach one that involved constantly readjusting programs rather than pre-planned periodization [8–10].

The previous statement can be seen as relatively broad and general, therefore coaches were presented with a more specific statement about whether training targets (within a specific training period) should remain fixed. Interestingly only 12% of periodization users (10% of all coaches) agreed, clearly conflicting with Cunanan et al. assertion that “once planned, fitness phases and other respective timelines are largely static” [4]. Following from the idea that changing the plan while acceptable is not desirable, we asked coaches whether consistently changing the plan was an indication of poor planning. Perhaps surprisingly among those who use periodization 61% disagreed with only 17% agreeing, this difference then only increased among those not using periodization (71% and 10%).

A fundamental characteristic of periodization is that once planned elements such as fitness phases should not need to be changed. This is predicated on the notion that training phases should take place in a pre-determined order. The responses in this section suggest that many of the surveyed coaches do not agree with this, instead adopting a dynamic approach aligned with the recommendations of the wider literature. Given the uncertainty inherent in the training process a flexible methodology which promotes persistent incremental adaptations, based on feedback, to both the goal and length of each training period is advised [42, 53]. Approaches analogous to this have been proposed in the training literature, with extensive theoretical rationales given, and have been chosen by the majority of coaches over periodization in several prior surveys [8–10, 15, 54]. Despite this, to date there seems to be little engagement with such an approach in experimental research let alone such thinking appearing as a viable alternative in educational material [5].

The world that periodization was born into is fundamentally different from todays. Research and coaches experience now suggest that approaches based upon predicting an athlete’s needs or the outcome of future training is unrealistic. Rather what is required is a planning approach that can adapt to ever-changing circumstances based on emerging information. Hence those working in this area of sports science need to engage with the coaching community to better understand how they have managed to navigate the pitfalls of periodization. Doing so would allow for more updated and relevant approaches to be presented in the literature and teaching curricula that they influence.

### Limitations

While this is possibly the first survey to examine coaches’ opinions on planning in such detail there are limitations that need to be acknowledged. As previously mentioned, probability sampling was not used in this study which carries certain limitations with it such as not being able to make statistical inferences. Therefore, the results from the current sample should not be generalized to the entire coaching population or specific sub-populations [16, 55]. It is worth noting though that the bar is quite high for statistical inferences to be made [56]. Furthermore, and reflecting a widespread gender bias within performance coaching, only 8% of respondents were female. Finally, this survey was advertised and delivered in English only leading it to be biased toward English speaking coaches. In part this led to an overrepresentation of the anglosphere in the demographic. While cultures effect on planning practices has not been established in sport cultural psychology and decision making research suggest it is possible [57, 58]. Unfortunately, due to the underrepresentation of coaches from outside of ‘Western’ cultures it is not possible to say whether this is the case in sport. It is important to note though that sampling is a problem that effects not just this study but many especially given the strict criteria for probability sampling.

Despite these limitations surveys like this do have strengths. For instance, this surveys novelty and exploratory nature give insight into some coaches’ perspectives on important topics surrounding the planning of training. Similar research would allow for greater understanding of important topics such as how coaches interpret other topics within the literature, how they test recommendations made and finally how they may determine their utility. Obviously due to the exploratory nature of this current survey further work is needed. With no surveys discussing these concepts the questions were specifically customized bringing both strengths and weaknesses. While the bespoke questions provided novel insights, some of the questions could be strengthened by an increase in detail or follow up questions. These would be worthwhile pursuing in future research. Similarly, and inevitably, despite striving for clear expression, and the use of piloting, some questions may have been misinterpreted [55].

### Recommendations for Future Research

In this survey 29% of coaches (with a range of experience and coaching levels) self-identified as non-periodization users. Nevertheless, within the literature, there are few planning methodologies offered as alternatives to periodization [59]. It remains unclear how non-periodizing coaches formulate, manage and deliver training plans, therefore future descriptive research could look to understand this. Indeed, some who did not self-describe as using periodization did use some concepts related with it, consequently a related question for these coaches is what are the boundaries of periodization. For those who self-describe as periodization users, but do not necessarily agree with statements taken from the theory, it would be useful to explore their specific interpretation of the term ‘periodization’. Lastly, for these coaches it would be useful to explore how they use periodization theory to aid decision-making processes.

A key takeaway from this survey is that there are coaches who deploy some, but not all, periodization theory directives as described in the literature. Furthermore, there seems a clear disconnect between coaches’ perspectives and practices and key periodization pillars as presented in the academic literature. These evident discrepancies indicate a need for closer collaboration between researchers and practitioners. An alternative research model such as integrated knowledge translation might be of assistance. The model involves knowledge users (coaches) having a meaningful partnership with a research team and being involved from study conception to application and publication [60]. This would be a shift from solely scientist driven research to a collaborative approach based on solving problems via consensus; thereby generating more contextually-sensitive and pragmatic solutions [61].

## Conclusion

The existing periodization literature, and many coach education initiatives, present periodization as the logical and best planning option for coaches. Indeed this perspective is echoed by one of the foremost experts on periodization Tudor Bompa, often referred to as a ‘father of periodisation’, when he stated “we either have periodization or chaos!” [62]. Given periodization’s presentation then as the primary approach to planning, in this study coaches were presented with a series of statements based on ‘core conventional training planning concepts’, as found in the literature, and asked to respond to them. The results of this survey illustrate discrepancies between coaches’ perspectives and key periodization principles. This was evident even among self-identified periodization users, where just over a third agreed with three fundamental periodization concepts. Given the results of this survey it appears many self-described periodization users are simply using modern planning best practices, rather than explicitly following periodization guidelines. Taken together these results suggest that, although periodization is the preeminent training planning approach represented in the literature, its principles are neither universally adhered to, nor pervasively accepted.

### Supplementary Information

**Additional file 1.** CHERRIES.

**Additional file 2.** Survey questions.

**Additional file 3.** Survey responses.

## Data Availability

Add data is stored and secured on the primary investigators institutional Microsoft OneDrive. Data is available on reasonable request.

## Abbreviation

SCC: Strength and conditioning coaches
