# Supplementary material for: Coaches’ Perceptions of Common Planning Concepts Within Training Theory: An International Survey
Source: Sports Med Open. 2023 Nov 21;9:109. doi: 10.1186/s40798-023-00657-6 (PMC10663426; doi:10.1186/s40798-023-00657-6)
Supplement: Supplementary file 1 — Additional file 1. CHERRIES. [file 40798_2023_657_MOESM1_ESM.docx]

**Checklist for Reporting Results of Internet E-Surveys (CHERRIES)**

| ***Checklist Item*** | ***Explanation*** |
| --- | --- |
| Describe survey design | It was an open survey. The target population were sport coaches who spoke English and were 18 years or older. A purposive convenient sample was used for the study. |
| IRB approval | Approved by lead authors IRB German Sport University Cologne ethics committee (Ethical Proposal Code 136/2021) |
| Informed consent | To participate in the survey participants were required to indicate that they had read the studies information sheet and were giving their informed consent. Participants were informed about which data would be stored, that it would be secured against access from those not involved in the study, that it would deanonymized, who the investigators were and the purpose of the study. |
| Data protection | Participants personal information was stored and secured on the primary investigators institutional Microsoft OneDrive with a backup kept on an external hard drive. Only the primary investigator had access to the participants personal information. |
| Development and testing | The initial survey was developed by the primary author with input from the final author. Content validity was initially established using external academic-coaches who have knowledge of the surveys area of interest. Feedback given was worked into the survey with a final round of content validity and piloting performed with a small group of experienced coaches. |
| Open survey versus closed survey | An open survey was used. |
| Contact mode | Survey was advertised on social media with link to survey. Emails were then sent to authors contacts who were asked to pass the link on to their own contacts (snowball) |
| Advertising the survey | Survey was initially advertised via social media. |
| Web/E-mail | The survey was stored as a Microsoft Forms document, with automatic method for capturing responses in the database. |
| Context | The survey was a simple Microsoft Forms document. The survey could be accessed by anyone with the link. |
| Mandatory/voluntary | It was a voluntary survey. |
| Incentives | No incentives were used. |
| Time/Date | November 2021 to February 2022 |
| Randomization of items or questionnaires | For the purpose of this study no randomization was needed. |
| Adaptive questioning | No adaptive questioning was used. |
| Number of Items | There were 14 items in total for this survey presented in this study. |
| Number of screens (pages) | 13 pages |
| Completeness check | All the items, aside for a question that gave the option of giving feedback at the end of the survey, were considered mandatory and therefore participants could not proceed without giving an answer to a question. |
| Review step | Participants were able to review and change their answers through the use of a back button. |
| Unique site visitor | N/A |
| View rate (Ratio of unique survey visitors/unique site visitors) | N/A |
| Participation rate (Ratio of unique visitors who agreed to participate/unique first survey page visitors) | 108 participants started the process of which 106 gave consent (98% of answer rate) |
| Completion rate (Ratio of users who finished the survey/users who agreed to participate) | 100% |
| Cookies used | No cookies were used. |
| IP check | IP addresses were not checked. |
| Log file analysis | N/A |
| Registration | N/A |
| Handling of incomplete questionnaires | No incomplete surveys were submitted. |
| Questionnaires submitted with an atypical timestamp | Participants were allowed as long as they wanted to fill out the survey. |
| Statistical correction | N/A |

This checklist has been modified from Eysenbach G. Improving the quality of Web surveys: the Checklist for Reporting Results of Internet E-Surveys (CHERRIES). J Med Internet Res. 2004 Sep 29;6(3):e34.
