# Supplementary material for: Coaches’ Perceptions of Common Planning Concepts Within Training Theory: An International Survey
Source: Sports Med Open. 2023 Nov 21;9:109. doi: 10.1186/s40798-023-00657-6 (PMC10663426; doi:10.1186/s40798-023-00657-6)
Supplement: Supplementary file 2 — Additional file 2. Survey questions. [file 40798_2023_657_MOESM2_ESM.docx]

Journal:

Sports Medicine – Open

Title:

Coaches’ perceptions of common planning concepts within training theory: An international survey

Author information:

Author 1

Kechi Anyadike-Danes (corresponding author)

Email: [kechi.anyadike-danes@stud.dshs-koeln.de](mailto:kechi.anyadike-danes@stud.dshs-koeln.de)

ORCID: 0000-0002-6393-966X

Department of Intervention Research in Exercise Training, German Sport University Cologne, Cologne, Germany

Author 2

Lars Donath

Department of Intervention Research in Exercise Training, German Sport University Cologne, Cologne, Germany

Author 3

John Kiely

Faculty of Education and Health Sciences, University of Limerick, Limerick, Ireland

| Would you describe your planning approach as training periodisation? | Yes  No |
| --- | --- |
| Do you see a distinction between periodisation and planning? | Yes  No |

| Please rate the extent to which you agree or disagree with the following statements | |
| --- | --- |
| Each season I evaluate my athletes needs. | Strongly disagree  Disagree  Neutral  Agree  Strongly agree |
| A goal is determined for each season. | Strongly disagree  Disagree  Neutral  Agree  Strongly agree |
| I divide the season into distinct manageable periods of time. | Strongly disagree  Disagree  Neutral  Agree  Strongly agree |
| At the beginning of the season defined and detailed goals are assigned to each distinct separate period of time. | Strongly disagree  Disagree  Neutral  Agree  Strongly agree |
| Physical capacities (e.g. strength, power) should be trained in a sequential order. | Strongly disagree  Disagree  Neutral  Agree  Strongly agree |
| Each training period should primarily focus on a specific physical capacity (for example: maximal strength). | Strongly disagree  Disagree  Neutral  Agree  Strongly agree |
| Physical adaptations are achievable within specific and fixed timeframes. | Strongly disagree  Disagree  Neutral  Agree  Strongly agree |
| Over the course of a competitive year, training should progress from general to  specific. | Strongly disagree  Disagree  Neutral  Agree  Strongly agree |
| Successfully completing the training plan optimizes the likelihood of the athlete achieving their pre-determined goals. | Strongly disagree  Disagree  Neutral  Agree  Strongly agree |
| Over the course of a training period, training targets should remain fixed. | Strongly disagree  Disagree  Neutral  Agree  Strongly agree |
| It is important to stick to the plan. | Strongly disagree  Disagree  Neutral  Agree  Strongly agree |
| Consistently changing the plan is a sign of poor planning. | Strongly disagree  Disagree  Neutral  Agree  Strongly agree |
